# A20 regulates IL-1-induced tolerant production of CXC chemokines in human mesangial cells via inhibition of MAPK signaling

**DOI:** 10.1038/srep18007

**Published:** 2015-12-09

**Authors:** Hongbo Luo, Yuming Liu, Qian Li, Lingjuan Liao, Ruili Sun, Xueting Liu, Manli Jiang, Jinyue Hu

**Affiliations:** 1 Medical Research Center, Changsha Central Hospital, Changsha, 410004, China; 2 Department of Urology, Renmin Hospital of Wuhan University, Wuhan 430060, China; 3 Department of Urology, Changsha Central Hospital, Changsha, 410004, China; 4 Department of Nephrology, Changsha Central Hospital, Changsha, 410004, China; 5 Department of Laboratory Medicine, XinXiang Medical University, XinXiang, 453003, China

## Abstract

Chemokines and chemokine receptors are involved in the resolution or progression of renal diseases. Locally secreted chemokines mediated leukocyte recruitment during the initiation and amplification phase of renal inflammation. However, the regulation of chemokine induction is not fully understood. In this study, we found that IL-1 induced a significant up-regulation of CXC chemokines CXCL1, 2, and 8 at both mRNA and protein levels in human mesangial cells. The induction of chemokines was tolerant, as the pre-treatment of HMC with IL-1 down-regulated the induction of chemokines induced by IL-1 re-stimulation. IL-1 up-regulated the ubiquintin-editing enzyme A20. A20 over-expression down-regulated IL-1-induced up-regulation of chemokines, and A20 down-regulation reversed chemokine inhibition induced by IL-1 pre-treatment, suggested that A20 played important roles in the tolerant production of chemokines. Unexpectedly, A20 over- expression inhibited the activation of ERK, JNK, and P38, but did not inhibit the activation of NF-κB. In addition, both IL-1 treatment and A20 over-expression induced the degradation of IRAK1, an important adaptor for IL-1R1 signaling, and A20 inhibition by RNA interference partly reversed the degradation of IRAK1. Taken together, IL-1-induced A20 negatively regulated chemokine production, suggesting that A20 may be an important target for the prevention and control of kidney inflammation.

The major problem in nephrology is the progression of the various renal disorders to the end-stage renal diseases. Only parts of renal diseases resolve after an acute phase whereas most tend to become chronic. Various kinds of injuries in kidney induce the inflammatory response, leading to the renal fibrosis, and the continuous decline of renal function. The progressive interstitial fibrosis is characterized by the infiltration of leukocytes and fibroblasts, and the accumulation of extracellular fibrous matrix. Chemokines and chemokine receptors have been reported to play critical roles in the infiltration of leukocytes and fibroblasts^1^. Chemokines function to mediate the infiltration of leukocytes during the initiation and amplification phase of renal inflammation. Subsequently, a rapid down-regulation of chemokines will support the resolution of acute inflammation, whereas the inflammation will progress if ongoing or repeated renal injury maintains to stimulate the local chemokine secretion and leukocyte influx into the glomerulus or the interstitial space^1^. Therefore, the understanding of the mechanism for chemokine production and their regulation in kidney is important, and may offer new therapeutic targets for the control of the progression of renal diseases.

Chemokines are small proteins which belong to the chemoattractant cytokine family^2^. By binding of chemokine receptors, a family of seven-transmembrane G protein-coupled receptors, chemokines play pivotal roles in controlling phagocyte recruitment during inflammatory responses^2^. Chemokines are also chemotactic for subsets of lymphocytes or dendritic cells to mediate acquired immunity^2^. In addition, chemokines have been reported to direct the migration of multipotent stem cells, and progenitor cells under physiologic and pathologic conditions, and play important roles in embryonic development, and tissue regeneration^3^.

Chemokines are up-regulated by a lot of pro-inflammatory cytokines, such as TNF-α, IL-6, IFN-α, IL-1β, and IL-1α^4^,^5^,^6^,^7^,^8^,^9^,^10^. Toll-like receptors also function to induce the production of chemokines^11^,^12^,^13^,^14^. However, the mechanism for the down-regulation and the termination of chemokine production after an initial stimulation by IL-1β is not fully understood. A20 (also known as TNFAIP3, tumor necrosis factor alpha-induced protein 3) is a potent anti-inflammatory signaling molecule that restricts multiple intracellular signaling cascades^15^. A20 has been reported to be up-regulated in endotoxin tolerance, be associated with the impaired LPS-induced signal transduction, and promote the induction of LPS tolerance^16^,^17^,^18^. By directly removing ubiquitin moieties from the signaling molecule TRAF6, A20 functions to terminate toll-like receptor-induced activity of the transcription factor NF-κB and pro-inflammatory gene expression in macrophages^19^. The similarity of signal transduction between toll-like-receptors and IL-1 receptors, such as the equal adaptor molecules MyD88 (myeloid differentiation factor 88), IRAK (Interleukin-1 receptor activated kinase)1, 2, 4, and TRAF6 (tumor necrosis factor receptor-associated factor 6)^20^,^21^, suggests that A20 may also function as negative regulator for chemokine production induced by IL-1. In this study, we found that the treatment of human mesangial cells with IL-1 induced a robust, but transient up-regulation of chemokine CXCL1, 2, and 8. IL-1-induced chemokines were regulated by A20 via inhibition of MAPK signaling.

## Results

### Human mesangial cells were important sources of CXC chemokines in response to IL-1

**Figure 1:**
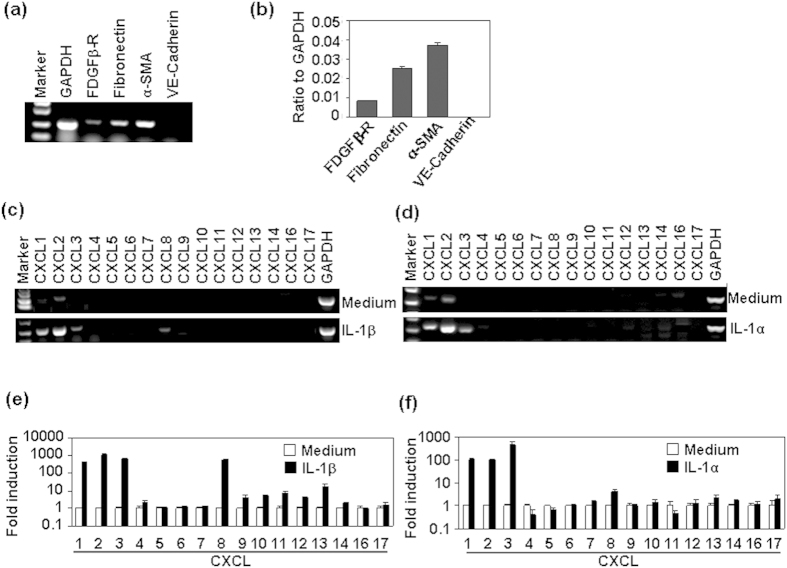
The effect of IL-1 on the mRNA expression of CXC chemokines. (**a**) The expression of mesangial cell makers in human mesangial cells. Cells, cultured in 6-well plate for 24 h, were harvested for the detection of mesangial cell markers by RT-PCR. GAPDH mRNA levels were detected as loading controls. (**b**) Quantitative dada for (**a**) by quantitative real time RT-PCR. (**c**,**d**) Human mesangial cells, treated with 20 ng/ml IL-1β (**c**), or IL-1α (**d**) for 1 h, were harvested for the detection of CXC chemokines by RT-PCR. GAPDH mRNA levels were detected as loading controls. (**e**) Quantitative data for (**c**) by real time RT-PCR. (**f**) Quantitative data for (**d**) by real time RT-PCR.

**Figure 2:**
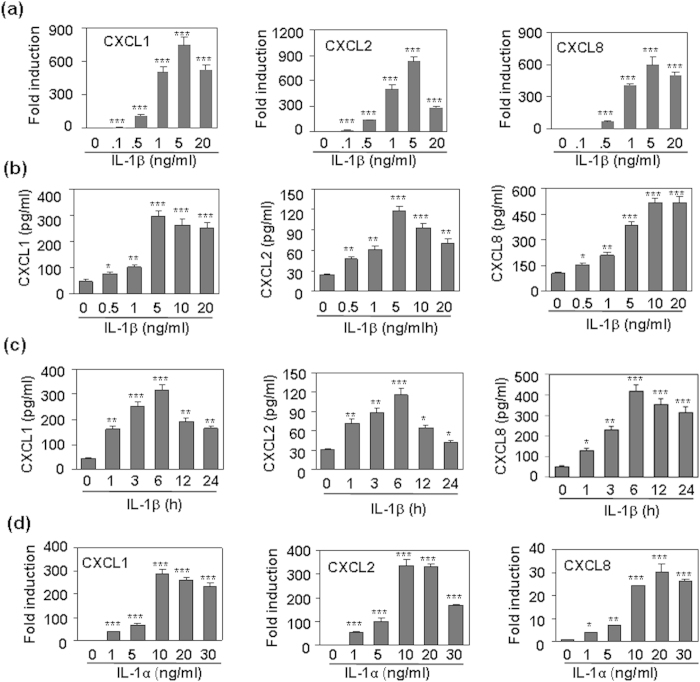
The dose- and time-dependent regulation of CXCL1, 2, and 8 induced by IL-1. (**a**) IL-1β dose-dependent regulation of the mRNA levels of chemokine CXCL1, 2, and 8. Human mesangial cells were treated with the indicated concentrations of IL-1β for 1 h. The mRNA levels of chemokine CXCL1, 2, and 8 were detected by qRT-PCR. (**b**) IL-1β dose-dependent regulation of the protein levels of chemokine CXCL1, 2, and 8. Human mesangial cells were treated with the indicated concentrations of IL-1β for 6 h. The chemokine protein levels in the supernatant were measured by ELISA. (**c**) IL-1β (10 ng/ml) time-dependent regulation of the protein levels of chemokine CXCL1, 2, and 8. (**d**) IL-1α dose-dependent regulation of the mRNA levels of chemokine CXCL1, 2, and 8. p-value *< 0.05, **< 0.01, and ***< 0.001 compared with the control groups.

Most types of intrinsic renal cells can secrete chemokines in response to immunologic, toxic, ischemic or mechanical injury^1^. IL-1 and its receptor IL-1R1 have been reported to play important roles in the induction of chemokine in alveolar epithelial cells, and in the subsequent inflammatory cell infiltration in ischemia-reperfusion injury^22^. In this study, we first detected the mesangial cell markers in human mesangial cells. Both RT-PCR and quantitative real time RT-PCR (qRT-PCR) results showed that human mesangial cells expressed mesangial cell markers, including platelet-derived growth factor β- receptor (PDGFβ-R), α-smooth muscle actin (α-SMA), and Fibronectin, but did not express endothelial cell marker VE-Cadherin (Fig 1a,b). Then we detected the effect of IL-1 on the expression of all CXC chemokines in human mesangeal cells, RT-PCR results showed that 20 ng/ml IL-1β or IL-1α induced up-regulation of CXCL1, 2, 3, and 8 (Fig. 1c,d). qRT-PCR results showed that IL-1 treatment led to an exaggerated induction of CXCL1, 2, 3, and 8 (Fig. 1e,f). Next, the dose- and time-dependent induction of CXCL1, 2, and 8 was analyzed. qRT-PCR results showed that 0.5–20 ng/ml IL-1β induced the up-regulation of CXCL1, 2, and 8, and the peak induction of chemokine was at 5 ng/ml (Fig. 2a). Meanwhile, ELISA results showed that the protein levels of CXCL1, 2, and 8 in the culture supernatant was significantly up-regulated in a dose- and time-dependent manner by the treatment of HMC cells with IL-1β (Fig. 2b,c). In addition, IL-1α also up-regulated the mRNA levels of CXCL1, 2, and 8 in a dose-dependent manner (Fig. 2d). These results suggested that HMC cells were important sources of CXCL1, 2, and 8 in response to IL-1.

### IL-1 pre-treatment induced a tolerance to the subsequent IL-1 re-stimulation

**Figure 3:**
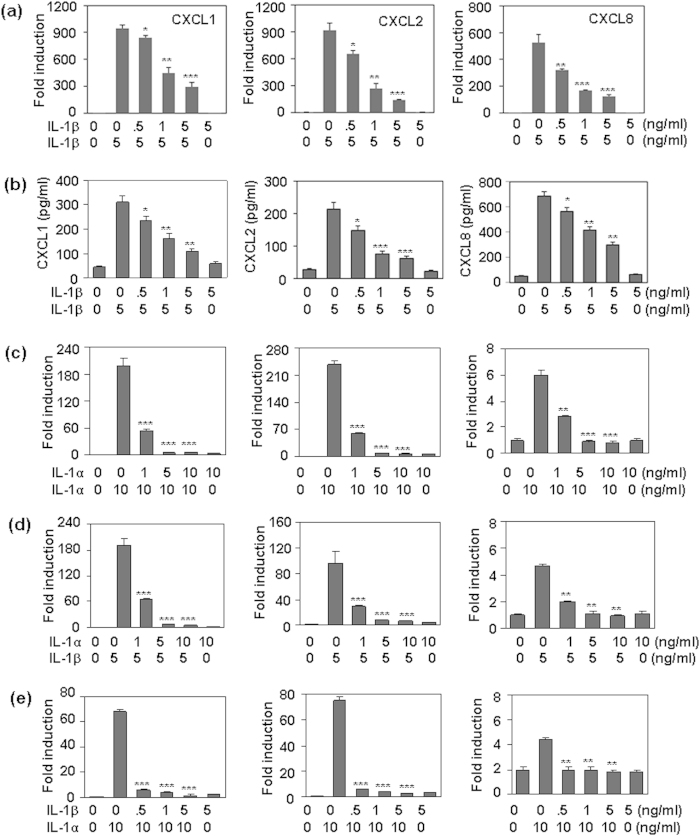
The effect of IL-1 pre-treatment on the production of CXCL1, 2, and 8 induced by IL-1 re-stimulation. (**a**) The effect of IL-1β pre-treatment on the mRNA production of CXCL1, 2, and 8 induced by IL-1β re-stimulation. HMC cells, pre-treated with the indicated concentrations of IL-1β for 24 h, were re-stimulated with 5 ng/ml IL-1β for 1 h. The mRNA levels of CXCL1, 2, and 8 were detected by qRT-PCR. (**b**) The effect of IL-1β pre-treatment on the protein induction of CXCL1, 2, and 8 induced by IL-1β re-stimulation. HMC cells, pre-treated with the indicated concentrations of IL-1β for 24 h, were re-treated with 5 ng/ml IL-1β for 6 h. The protein levels of CXCL1, 2, and 8 were detected by ELISA. (**c**) The effect of IL-1α pre-treatment on the mRNA production of CXCL1, 2, and 8 induced by IL-1α re-stimulation. (**d**) The effect of IL-1α pre-treatment on the protein induction of CXCL1, 2, and 8 induced by IL-1β re-stimulation. (**e**) The effect of IL-1β pre-treatment on the protein induction of CXCL1, 2, and 8 induced by IL-1α re-stimulation. p-value *< 0.05, **< 0.01, and ***< 0.001 compared with the IL-1-treated alone groups.

In professional immune cells, Toll-like receptor 4 (TLR4) induces tightly regulated inflammatory response to avoid tissue damage via the induction of “endotoxin tolerance”, which is a transient state of cell desensitization in response to LPS re-stimulation after a prior LPS exposure. To confirm whether the CXC chemokine induction was tolerant, we first detected the effect of IL-1β pre-treatment on the chemokine production induced by IL-1β re-stimulation. Both qRT-PCR and ELISA results showed that the pre-treatment of HMC cells with 0.5–5 ng/ml IL-1β significantly reversed the production of CXCL1, 2, and 8 induced by the re-stimulation of 5 ng/ml IL-1β at both mRNA and protein levels (Fig. 3a,b). Chemokine tolerant induction was also observed when the HMC cells, pre-treated with IL-1α, were re-stimulated with IL-1α (Fig. 3c). Meanwhile, cross-tolerant induction of chemokines was also found when cells, pre-treated with IL-1α, were re-stimulated with IL-1β (Fig. 3d), and when cells, pre-treated with IL-1β, were re-stimulated with IL-1α (Fig. 3e). These results suggested that IL-1 induced a tolerant production of CXCL1, 2, and 8 in human mesangial cells.

### IL-1 pre-treatment down-regulated MAPK and NF-κB signaling induced by IL-1 re-stimulation

**Figure 4:**
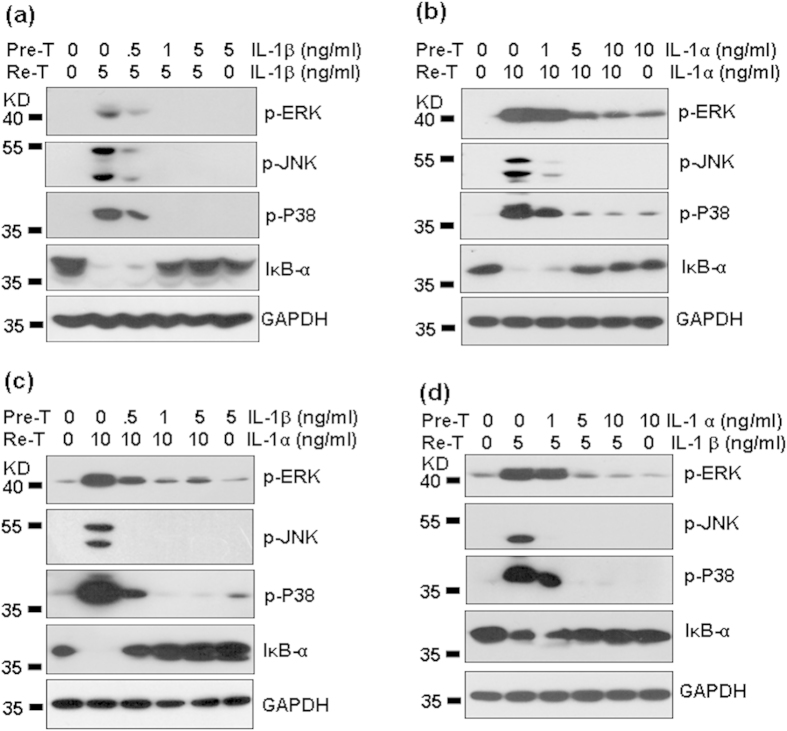
The effect of IL-1 pre-treatment on the activation of MAPKs and NF-κB induced by IL-1 re-stimulation. (**a**) The effect of IL-1β pre-treatment on the activation of MAPKs and NF-κB induced by IL-1β re-stimulation. HMC cells, pre-treated with the indicated concentrations of IL-1β for 24 h, were re-treated with 5 mg/ml IL-1β for 30 min. The phosphorylation of MAPKs ERK (42/44 KD), JNK (46/54 KD), and p38 (38 KD), and the total levels of IκB-α (39 KD) were detected by western blot. GAPDH (36 KD) protein levels were detected as loading controls. (**b**) The effect of IL-1α pre-treatment on the activation of MAPKs and NF-κB induced by IL-1α re-stimulation. (**c**) The effect of IL-1β pre-treatment on the activation of MAPKs and NF-κB induced by IL-1α re-stimulation. (**d**) The effect of IL-1α pre-treatment on the activation of MAPKs and NF-κB induced by IL-1β re-stimulation.

Activation of the MAPKs and NF-κB is important in the production of pro-inflammatory cytokines. The inhibition of signal transduction contributes to the induction of endotoxin-tolerance. We detected the effect of IL-1 pre-treatment on the signaling transduction induced by IL-1 re-stimulation. Western blot results showed that the pre-treatment of HMC cells with 0.5–5 ng/ml IL-1β significantly reversed the activation of MAPKs P38, JNK, and ERK (Fig. 4a), and the degradation of IκB-α (Fig. 4a) induced by the re-stimulation of 5 ng/ml IL-1β. Meanwhile, pre-treatment of HMC cells with IL-1α also inhibited the activation of MAPKs and NF-κB (Fig. 4b) induced by IL-1α re-stimulation. Cross-inhibition of MAPK and NF-κB signaling between IL-1α and IL-1β was also found (Fig. 4c,d). These results suggested that IL-1-induced tolerance may be due to the inhibition of signaling transduction by IL-1 pre-treatment.

### The effect of IL-1R2 and IL-1Ra on the tolerance production of chemokines

**Figure 5:**
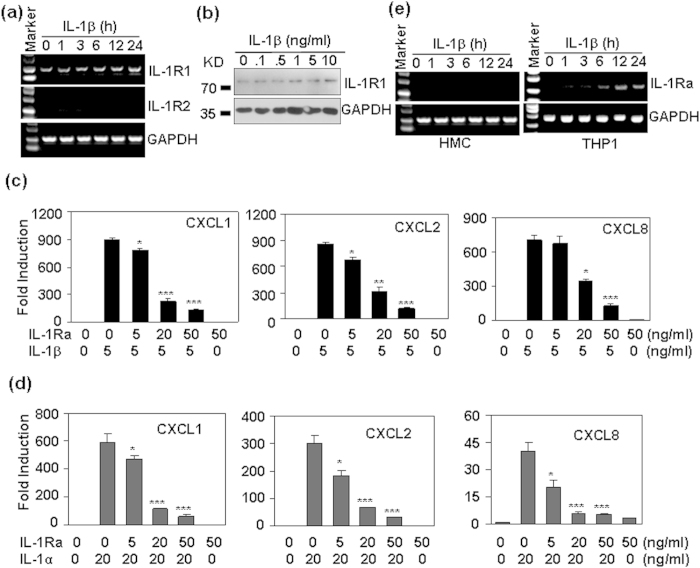
The effect of IL-1R2 and IL-1Ra on IL-1-induced chemokine production. (**a**) The effect of IL-1β on the mRNA levels of IL-1 receptors. HMC cells were treated with 20 ng/ml IL-1β for the indicated time periods, the mRNA levels of IL-1R1, and IL-1R2 were detected by RT-PCR. GAPDH mRNA levels were detected as loading controls. (**b**) The effect of IL-1β on the protein levels of IL-1R1. HMC cells were treated with the indicated concentrations of IL-1β for 24 h. The protein levels of IL-1R1 (80 KD) were detected by western blot. GAPDH (36 KD) protein levels were detected as loading controls. (**c**) The effect of IL-1Ra on the mRNA levels of CXCL1, 2, and 8 induced by IL-1β. HMC cells, pre-treated with the indicated concentrations of IL-1Ra for 1 h, were re-stimulated with 5 ng/ml IL-1β for 1 h. The mRNA levels were detected by qRT-PCR. (**d**) The effect of IL-1Ra on the mRNA levels of CXCL1, 2, and 8 induced by IL-1α. (**e**) The effect of IL-1β on the mRNA levels of IL-1Ra in HMC cells and in human monocytic THP-1 cells. p-value *< 0.05, **< 0.01, and ***< 0.001 compared with the IL-1 treated alone groups.

IL-1 receptor 2 (IL-1R2) acts as a decoy receptor for IL-1^23^, and IL-1 receptor antagonist (IL-1Ra) functions to inhibit IL-1R1 activation by inhibition of IL-1α and β binding with IL-1R1. To confirm whether the chemokine tolerance was related to the modulation of IL-1R2 or IL-1Ra, the expression of IL-1 receptors IL-1R1, and 2 were detected first. RT-PCR results showed that HMC cells expressed only IL-1R1, but not IL-1R2 (Fig. 5a). The treatment of HMC cells with IL-1β did not induce significant modulation of IL-1R1 and IL-1R2 (Fig. 5a). IL-1R1 protein was not significantly regulated either (Fig. 5b). When HMC cells, pre-treated with IL-1Ra, an IL-1R antagonist, were re-stimulated with IL-1β, we found that the up-regulation of CXCL1, 2 and 8 was reversed (Fig. 5c). Similarly, the pre-treatment of HMC cells with IL-1Ra also reversed the up-regulation of CXCL1, 2, and 8 induced by IL-1α (Fig. 5d). However, IL-1 did not induce the production of IL-1Ra in HMC cells (Fig. 5e). As a positive control, IL-1 induced the expression of IL-1Ra in monocytic THP-1 cells (Fig. 5e). These results suggested that both IL-1R2 and IL-1Ra did not contribute to the induction of chemokine tolerance in human mesengial cells.

### IL-1 induced the expression of the ubiquitin-editing enzyme A20

**Figure 6:**
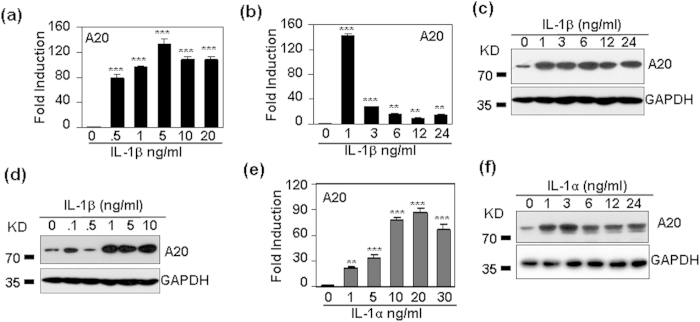
IL-1 dose- and time-dependently regulates A20 expressopm in human mesangial cells. (**a**) IL-1β dose-dependent regulation of A20 mRNA. HMC cells were treated with indicated concentrations of IL-1β for 1 h. The mRNA levels of A20 were detected by qRT-PCR. (**b**) IL-1β time-dependent regulation of A20 mRNA. HMC cells were treated with 5 ng/ml IL-1β for the indicated time periods. The mRNA levels of A20 were detected by qRT-PCR. (**c**) IL-1β time-dependent regulation of A20 (82 KD) protein. (**d**) IL-1β dose-dependent regulation of A20 protein. (**e**) IL-1α dose-dependent regulation of A20 mRNA. (**f**) IL-1α time-dependent regulation of A20 protein. p-value **< 0.01, and ***< 0.001 compared with the control groups.

The ubiquitin-editing enzyme A20 (also named tumor necrosis factor alpha-induced protein 3, TNFAIP3), has been reported to be up-regulated in endotoxin tolerance, to be associated with the impaired LPS-induced signal transduction, and to promote the induction of LPS tolerance (16–18). To determine whether A20 is involved in the induction of IL-1 tolerance in human mesangial cells, A20 expression after IL-1 treatment was tested by qRT-PCR and western blot. The results showed that 0.5–20 ng/ml IL-1β significantly up-regulated mRNA levels of A20 (Fig. 6a), and A20 mRNA was rapidly induced after IL-1β stimulation, with the peak induction at 1 h (Fig. 6b). Meanwhile, A20 protein levels were also dose- and time-dependently up-regulated (Fig. 6c,d). When HMC cells were treated with IL-1α, A20 was also induced dose-dependently and rapidly (Fig. 6e,f).

### The effect of A20 over-expression on IL-1-induced chemokine production

**Figure 7:**
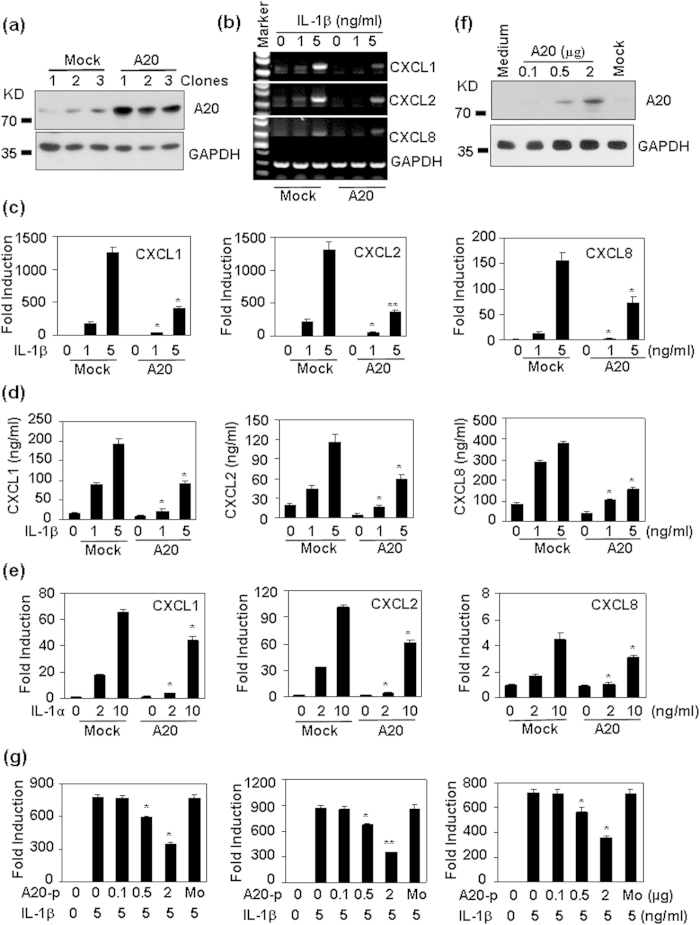
The over-expression of A20 inhibits IL-1-induced chemokines. (**a**) A20 protein levels in Mock- and A20-transfected cell clones. HMC cells were mock-tranfected, or transfected with A20 mammalian expressing plasmid, G418-resistant clones were selected and A20 (82 KD) protein levels were detected by western blot. GAPDH (36KD) protein levels were detected as loading controls. (**b**) The effect of A20 over-expression on IL-1β-induced mRNA expression of CXCLs. Mock- and A20-transfected cells were treated with the indicated concentrations of IL-1β for 1 h. The mRNA levels of CXCL1, 2, and 8 were detected by RT-PCR. GAPDH mRNA levels were detected as loading controls. (**c**) The quantitative data for (**b**). (**d**) The effect of A20 over-expression on IL-1β-induced protein expression of CXCLs. Mock- and A20-transfected cells were treated with the indicated concentrations of IL-1β for 6 h. The protein levels of CXCL1, 2, and 8 in the culture supernatant were detected by western blot. GAPDH protein levels were detected as loading controls. (**e**) The effect of A20 over-expression on IL-1α-induced mRNA expression of CXCLs. p-value *< 0.05, and **< 0.01 compared with mock-transfected groups. (**f**) The effect of A20 transient transfection on A20 expression. Cells were transfected with indicated A20 mammalian expression plasmid. 48 h after transfection, the expression levels of A20 were detected by western blot. GAPDH protein levels were detected as loading controls. (**g**) The effect of A20 transient transfection on IL-1β-induced chemokine expression. Cells were transiently transfected with indicated A20 mammalian expression plasmid. 48 h after transfection, the cells were treated with 5 ng/ml IL-1β for 1 h. CXCL1, 2, and 8 mRNA levels were detected by qRT-PCR. p-value *< 0.05, and **< 0.01 compared with non-transfected, IL-1β-treated groups.

A20 functions to terminate Toll-like receptor (TLR)-induced immune response^19^, and plays important roles in the induction of endotoxin-tolerance^24^, and Pam3CSK4- tolerance^25^. To confirm the involvement of A20 in the regulation of chemokine production, we transfected HMC cells with A20 mammalian expressing vector. Western blot results showed that A20 expression was increased in three G418-resistante clones compared with that in three mock-transfected clones (Fig. 7a). When A20 over-expressed cells were treated with IL-1β, RT-PCR results showed that the up-regulation of CXCL1, 2, and 8 was decreased compared with that induced in mock-transfected cells (Fig. 7b). qRT-PCR results showed that A20 over-expression-induced down-regulation of CXCL1, 2, and 8 mRNA was significant (Fig. 7c). Meanwhile, A20 over-expression also significantly reversed IL-1β-induced protein production of CXCL1, 2, and 8 in the culture supernatant (Fig. 7d). In addition, A20 partly reversed the induction of CXCL1, 2, and 8 induced by IL-1α (Fig. 7e). To determine the dose-dependent inhibition of chemokine production by A20, HMC cells were transiently tranfected with various doses of A20 plasmid. Western blot results showed that A20 protein levels were dose-dependent induced after A20 transient transfection (Fig. 7f). Accordingly, A20 dose-dependently inhibited Il-1β-induced CXCL1, 2, and 8 (Fig. 7g). These results suggested that A20 was an important regulator for IL-1-induced CXC chemokines in human mesangial cells.

### The effect of A20 down-expression on chemokine inhibition induced by IL-1 pre-treatment

**Figure 8:**
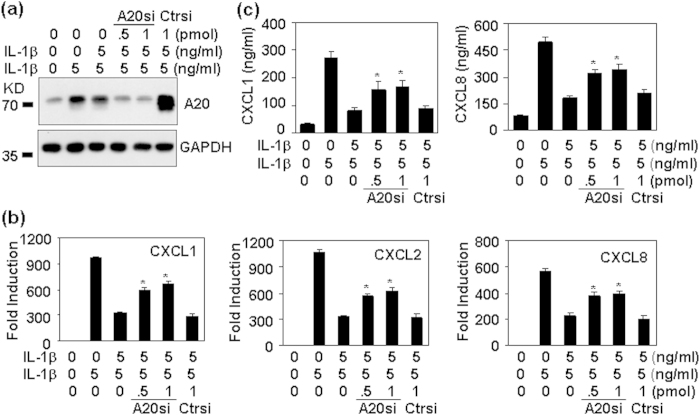
The down-expression of A20 reverses chemokine inhibition induced by IL-1 pre-treatment. HMC cells, tranfected with the indicated concentration of A20 siRNA (A20si), or with control siRNA (Ctrsi) for 5 h, were cultured for 12 h, followed by the pre-treatment with 5 ng/ml IL-1β for 24 h, and then a treatment with IL-1β for 2 h for A20 protein detection (**a**), for 1 h for chemokine mRNA detection (**b**), and for 6 h for chemokine protein detection (**c**). p-value *< 0.05, compared with non-transfected, IL-1β-pre-treated groups.

To further confirm the involvement of A20 in the regulation of chemokine production, we transfected HMC cells with A20 siRNA to down-regulate A20 expression. Western blot results showed that IL-1-induced A20 up-regulation was reversed in A20 siRNA-transfected cells (Fig. 8a). qRT-PCR and ELISA results showed that A20 down-regulation reversed chemokine inhibition induced by IL-1 pre-treatment in both mRNA levels (Fig. 8c) and protein levels (Fig. 8c). These results suggested that A20 involved in the tolerant production of IL-1-induced CXC chemokines in human mesangial cells.

### The effect of A20 over-expression on IL-1-induced signaling

**Figure 9:**
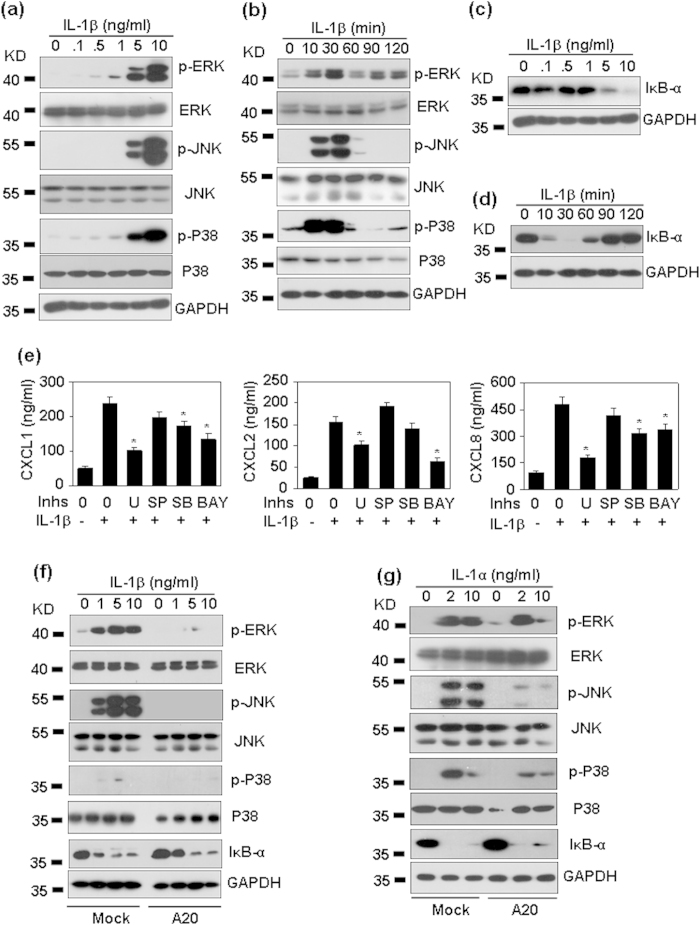
The signal transduction for CXC chemokine production induced by IL-1. (**a**) IL-1β dose dependent activation of MAPKs. HMC cells were treated with the indicated concentrations of IL-1β for 30 min. The phosphorylation of MAPKs ERK (42/44 KD), JNK 46/54 KD), and p38 (38 KD) was detected by western blot. GAPDH (36 KD) protein levels were detected as loading controls. (**b**) IL-1β time-dependent activation of MAPKs. HMC cells were treated with 10 ng/ml IL-1β for the indicated time periods. The phosphorylation of MAPKs ERK, JNK, and p38 was detected by western blot. GAPDH protein levels were detected as loading controls. (**c**) IL-1β dose-dependent degradation of IκB-α (39 KD). (**d**) IL-1β time-dependent degadation of IκB-α. (**e**) The effect of signal inhibitors on chemokine induction. HMC cells, pre-treated without medium, or ERK inhibitor U0126 (U, 10 μM), or JNK inhibitor SP600125 (SP, 10 μM), or P38 inhibitor SB203580 (SB, 10 μM), or NF-κB inhibitor Bay 117082 (Bay, 10 μM) for 30 min, were re-stimulated with 5 ng/ml IL-1β for 6 h. Chemokine protein levels in the culture supernatant were detected by ELISA. p-value *< 0.05 compared with the IL-1β-treated alone groups. (**f**) The effect of A20 over-expression on IL-1β-induced signal transduction. Mock- and A20-transfected HMC cells were treated with the indicated concentration of IL-1β for 30 min. The phosphorylation of MAPKs, and the total protein levels of IκB-α were detected as same as (**a**). (**g**) The effect of A20 over-expression on IL-1α-induced signal transduction. p-value *< 0.05 compared with the IL-1β-treated alone groups.

IL-1 prefers to activate MAPKs, including ERK, JNK and P38, and activate NF-κB. To determine whether A20 regulates IL-1-induced signaling, we first detected the phosphorylation of ERK, JNK and P38, and the degradation of IκB-α induced by IL-1β in human masengial cells. Western blot results showed that IL-1β dose- (Fig. 9a) and time-dependently (Fig. 9b) induced the phosphorylation of JNK, ERK, and P38, and the degradation of IκB-α (Fig. 9c,d). To determine which pathway was involved in the induction of chemokines, HMC cells, pre-treated with the inhibitors of ERK, JNK, P38, or NF-κB respectively, were re-stimulated with IL-1β, and the induction of chemokines was detected by qRT-PCR. The results showed that ERK and NF-κB pathways were involved in the production of CXCL1, 2, and 8 (Fig. 9e). P38 pathway was involved in the production of CXCL1, and 8 (Fig. 9e). To determine whether A20 regulates IL-1- induced signaling, A20 over-expressed cells were treated with IL-1β, and the activation of signaling was detected by western blot. The results showed that A20 over-expression significantly reversed IL-1β-induced phosphorylation of ERK, JNK, and P38 (Fig. 9f). To our surprise, A20 over-expression did not reverse IL-1β-induced degradation of IκB-α (Fig. 9f). Similarly, A20 over-expression significantly reversed IL-1α-induced phosphorylation of ERK, JNK, and p38, but did not affect IL-1α-induced degradation of IκB-α (Fig. 9g). These results suggested that A20 regulated IL-1-induced the chemokine production via inhibition of MAPK signal transduction.

### IL-1 treatment and A20 over-expression induced the degradation of IRAK1

**Figure 10:**
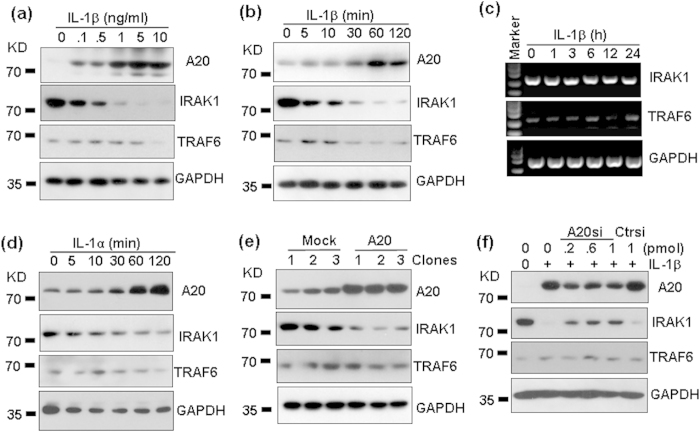
The effect of A20 on the expression of TRAF6 and IRAK1. (**a**) Dose dependent regulation of A20, IRAK1 and TRAF6 protein levels by IL-1β. HMC cells were treated with the indicated concentrations of IL-1β for 2 h. The protein levels of A20 (82 KD), IRAK1 (78 KD), and TRAF6 (60 KD) were detected by western blot. GAPDH (36 KD) protein levels were detected as loading controls. (**b**) Time-dependent regulation of A20, IRAK1, and TRAF6 protein levels by IL-1β. (**c**) Dose-dependent regulation of IRAK1 and TRAF6 mRNA levels by IL-1β. (**d**) Time dependent regulation of A20, IRAK1 and TRAF6 protein levels by IL-1α. (**e**) The effect of A20 over-expression on the protein levels of A20, IRAK1 and TRAF6. HMC cells, transfected with 2 μg plasmid containing the sequence coding for human A20 protein, or with empty plasmid, G418-resistant clones were selected for the detection of A20, IRAK1, and TRAF6 expression by western blot. GAPDH protein levels were detected as loading controls. (**f**) The effect of A20 down-regulation on the protein levels of A20, IRAK1, and TRAF6. HMC cells, transfected with the indicated concentrations of A20 siRNA, or control siRNA, for 5 h, were cultured for 36 h, followed by the treatment with 5 ng/ml IL-1β for 2 h. The protein levels of A20, IRAK1, and TRAF6 were detected by western blot. GAPDH protein levels were detected as loading controls.

IL-1R-associated kinase 1 (IRAK1) has been suggested to play crucial roles in endotoxin tolerance^26^,^27^,^28^. And IRAK1 degradation has also been reported to exert anti-inflammatory effect^29^,^30^. To determine whether A20-induced inhibition of chemokine expression and signal transduction were due to the degradation of IRAK1, the protein levels of IRAK1 in response to IL-1β treatment were detected by western blot. The results showed that rest HMC cells expressed low level of TRAF6, but high level of IRAK1 (Fig. 10a). IL-1β treatment induced a slight down-regulation of TRAF6 protein, but induced a strong down-regulation of IRAK1 protein in a dose and time dependent manner (Fig. 10a,b). However, IL-1β treatment did not induce the down-regulation of mRNAs of TRAF6 and IRAK1 (Fig. 10c). Meanwhile, IL-1α treatment also induced the down-regulation of IRAK1 protein (Fig. 10d). We also found that IL-1 treatment induced the up-regulation of A20 protein accordingly (Fig. 10a,b,d). To determine whether the up-regulation of A20 was associated with the down-regulation of IRAK1, HMC cells were transfected with A20, and IRAK1 protein levels were detected in three mock-transfected and three A20 over-expression clones. Western blot results showed that A20 over- expression by gene transfer induced a significant down-regulation of IRAK1, but TRAF6 levels were not significantly regulated (Fig. 10e). When HMC cells, transfected with A20 siRNA to inhibit A20 protein level, were treated with IL-1β, the up-regulation of A20 was inhibited (Fig. 10f). Accordingly, the down-regulation of IRAK1 induced by IL-1β was partly reversed (Fig. 10f). No significant modulation of TRAF6 was found (Fig. 10f). These results suggested that IL-1 induced the degradation of IRAK1 via up-regulation of A20.

## Discussion

Most human diseases are due to chronic inflammation, resulting in the loss of organ function. Interleukin-1 (IL-1) is a master cytokine of local and systemic inflammation. In the necrotic area of acute inflammation, dying cells lose membrane integrity, leading to the release of IL-1α, which activates nearby resident macrophages by binding to the IL-1RI to synthesize more cytokines including IL-1β^31^. Both IL-1α and IL-1β activate nearby resident fibroblasts, epithelial cells to release chemokines and establish a chemokine gradient to elicit the infiltration of neutrophils and monocytes, which function to scavenge dead cells and debris^31^. However, the excessive inflammatory responses elicit a secondary damage following a primary injury. Therefore, both IL-1 and IL-1 receptor are targets for the control of a broad spectrum of diseases^31^. In this study, we found that human mesangial cells expressed IL-1R1. The activation of IL-1R1 induced the phosphorylation of NF-κB and MAPKs. These results suggested that IL-1 gene cluster involves in the pathogenesis of renal diseases, and that the polymorphisms of IL-1 gene cluster may predict the risk of renal diseases^32^,^33^.

The course of progressive renal disorders can be divided into four phases: the initiation phase, the amplification phase, the progression phase, and the terminal phase. Pro-inflammatory cytokines induced by any type of renal parenchymal cells in response to the injuries elicit leukocyte infiltration and activation at multiple stages^1^. Meanwhile, chemokines and chemokine receptors have been reported to be involved in the progression of multiple renal diseases^33^,^34^. Blockade of CCR1, a chemokine receptor for several ligands, including CCL3, 5, 7, 8, 14, 15, 16, and 23^2^, by use of the antagonist BX471, was found to reduce cell accumulation and renal fibrosis in unilateral ureter obstruction^35^. Blockade of CCR2, a chemokine receptor for ligands CCL2, 7, 8, 13, and 16^2^, has been reported to improve insulin resistance, lipid metabolism, and diabetic nephropathy in type 2 diabetic mice^36^. Ccr2 deficient mice has been found to survive longer, develop less lymphadenopathy, less proteinuria, reduced lesion scores, and less infiltration by T cells and macrophages in the glomerular and tubulointerstitial compartments^37^. CXC chemokine receptor CXCR1 was found to be expressed in human glomerular diseases, including crescentic glomerulonephritis, immunoglobulin A nephropathy, membranoproliferative glomerulonephritis, lupus nephritis, membranous nephropathy, and non-involved parts of tumor nephrectomies^38^. CXCR2 knockout mice are protected against DSS-colitis-induced acute kidney injury and inflammation^39^. The functional polymerphisms of CXCL8 (also named interleukin -8, a ligand of CXCR1, and 2) is related to the susceptibility to acute pyelonephritis^40^. The activation of toll-like receptor 4 in intrinsic renal cells induced the antibody-mediated glomerulonephritis via CXCL1 and 2^41^. We found here that the activation of IL-1R1 in human mesangail cells induced a transient but robust up-regulation of CXCL1, 2, and 8, suggesting that IL-1 gene cluster may involve in the pathogenesis of renal diseases by induction of CXC chemokines.

In professional immune cells, Toll-like receptors induce tightly regulated inflammatory response to avoid tissue damage via the induction of tolerance, which is a transient state of cell desensitization in response to ligand re-stimulation after a prior ligand exposure (16-18, 27). In our study, we found that the pre-treatment of human mesangial cells with IL-1β reversed the chemokine production induced by IL-1β re-stimulation, suggesting that a tolerance also existed in the production of chemokines in response to IL-1β treatment. This phenomenon of the tolerance in chemokine production was consistent to the progression of renal diseases, in which, more chemokines were necessary for more immune cell infiltration in the initial and the amplification phases, but in the progression, and the terminal phases, chemokines need to be down-regulated (1).

A20 (also known as TNFAIP3) is a potent anti-inflammatory signaling molecule that restricts multiple intracellular signaling cascades. Human genetic studies have linked germline singlenucleotide polymorphisms (SNPs) of TNFAIP3 with susceptibility to multiple human diseases, including systemic lupus erythematosus (SLE), rheumatoid arthritis, psoriasis, type 1 diabetes, coeliac disease, Crohn’s disease, coronary artery disease in type 2 diabetes, and systemic sclerosis (15). Cell type-specific ablation of A20 expression in B cells, dendritic cells, macrophages, and intestinal epithelial cells, lead to the production of autoantibody, immune cell expansion and activation, colitis, nephritis, and infections (15). In our study, we found IL-1 treatment of human mesangial cells induced a robust and rapid production of A20 at both mRNA and protein levels. The over-expression of A20 inhibited IL-1-induced chemokine production, and inhibited IL-1-activated signal transduction. These results suggested that A20 functions as a regulator for leukocyte infiltration in local inflammatory tissues.

Toll-like receptors and IL-1 receptors elicit their signal transduction via their TIR (Toll/interleukin-1 receptor) domain. Binding of ligands to IL-1 receptor or toll-like receptors results in the recruitment of MyD88, followed by the activation of IRAKs and the E3 ubiquitin ligase TRAF6, and both IL-1 receptors and toll-like receptors finally activate MAPKs p38, JNK, and ERK, and NF-κB^20^,^42^. The activation of toll-like receptors elicits innate immune response. At the same time, toll-like receptors induce the expression of A20, which functions as a negative feedback regulator to terminate the immune responses induced by toll-like receptors^19^. A20 terminates NF-κB signaling by inhibition of the E3 ligase activities of TRAF6, TRAF2, and cIAP1 via antagonizing interactions with the E2 ubiquitin conjugating enzymes Ubc13 and UbcH5c^43^,^44^. A20 has also been reported to inhibit IL-1 signaling^45^,^46^. But the mechanism remains unclear. In this study, we found that IL-1 treatment of human mesangial cells induced the degradation of IRAK1, at the same time, A20 over-expression inhibited IL-1-induced signal transduction, and degraded the protein levels of IRAK1. Inhibition of A20 by RNA interference reversed IL-1β-induced degradation of IRAK1. These results suggested that IL-1-induced chemokines were regulated by A20 via degradation of IRAK1.

Early IRAK1 studies revealed that the activation of TLR/IL-1R signaling pathways induces rapid auto-phosphorylation of IRAK1 and a transient appearance of higher molecule weight forms of IRAK1 which are results of hyper-phosphorylation and ubiquitin modifications^47^. In our study, we found that A20 over-expression down-regulated IRAK1 at protein levels, but not at mRNA levels. So we guess that A20 may promote IRAK1 degradation by ubiquitination

A20 negatively regulates TLR-induced inflammatory response by inhibition of NF-κB signaling^43^. In our study, we found that A20 regulated IL-1-induced chemokine production. The over-expression of A20 inhibited the induction of CXC chemokines CXCL1, 2, and 8. Meanwhile, A20 over-expression inhibited the activation of MAPKs ERK, JNK, and p38, but did not inhibit the activation of NF-κB induced by IL-1^47^. In addition, A20 over-expression only partly reversed IL-1-induced production of chemokines. These results suggested that A20 regulation of IL-1R1 signaling is different from that of TLR signaling, and other mechanisms exist for the regulation of IL-1-induced production of CXC chemokines.

## Materials and Methods

### Cell lines and reagents

Human mesangial cells (HMC)^48^ were grown in DMEM containing 10% FCS, 100 units/ml penicillin, and 100 mg/ml streptomycin. Recombinant human IL-1α, IL-1β, IL-1Ra, human CXCL1, 2, 8 ELISA kits were purchased from PeproTech (Rocky Hill, NJ, USA). Mouse anti-human IL-1R1 antibody was purchased from Abcam (Cambridge, UK). Rabbit anti-human phosphorylated ERK, JNK, and p38 antibodies, rabbit anti-human A20 antibody, and ERK inhibitor U0126 were purchased from Cell Signaling Technology (Beverly, MA, USA). P38 inhibitor SB203580, JNK inhibitor SP600125, and NF-κB inhibitor Bay117082 were purchased from Tocris (Ellisville, MO, USA). A20 mammalian expressing plasmid (EX-K6040-M11) was purchased from Genecopoeia (Rockville, MD, USA).

### Reverse transcription-PCR (RT-PCR)

Total RNA was extracted from 1 to 2 × 10^6^ cells using TRIzol (Invitrogen, Carlsbad, CA, USA), as described by the manufacturer. mRNA was reverse transcribed with RevertAid (MBI Fermentas, Burlington Ontario, Canada) at 42 °C for 60 min, and the resulting cDNA was subjected to PCR (94 °C for 1 min followed by 20–25 cycles at 94 °C for 30 s, 60 °C for 30 s, and 68 °C for 1 min and an extension for 10 min at 68 °C). The PCR products were separated on 1.0% agarose gels and visualized with GelRed (Biotium). The forward and reverse primer pairs are listed (5′ to 3′) as follows:

A20-F: ATGAGGCCAAAAGGACAGAA

A20-R: ACTGAAAGCATTCGTTGCAG

GAPDH-F, AATCCCATCACCATCTTCCA,

GAPDH-R, CCTGCTTCACCACCTTCTTG;

IL-1R1-F, TGCCTGCTTGAAGGAACAGT

IL-1R1-R, ATTCTTGGTCATCATCACCCC

IL-1R2-F, TCCTGACATTTGCCCATGAA

IL-1R2-R, TTCTGAATATTCCTGGCGTG

IL-1Ra-F, CCAAATTCTACTTCCAGGAGG

IL-1Ra-R, TCGGGGTTTGATCCTGAAT

CXCL1-S: TCACCCCAAGAACATCCAAA

CXCL1-A: TCCTAAGCGATGCTCAAACA

CXCL2-S: GCAGGGAATTCACCTCAAGAA

CXCL2-A: AACACATTAGGCGCAATCCA

CXCL3-S: AACAGCAGCTTTCTAGGGACA

CXCL3-A: GTGATCCACTAATTGCTTGCA

CXCL4-S: AGTCATTGGCCACAGAGACC

CXCL4-A: TTCCTTCCATTCTTCAGCGT

CXCL5-S: ACAGGCAAATTCCTGACTGCT

CXCL5-A: CCATAAATGCTGGCCTTCTT

CXCL6-S: ACGCTGAGAGTAAACCCCAAA

CXCL6-A: TTTCCCCCACACTCTTCAAA

CXCL7-S: ACACTGAAGGATGGGAGGAAA

CXCL7-A: GGGTTGAAACCAGGCTTATT

CXCL8-S: TTGGCAGCCTTCCTGATTT

CXCL8-a: TCAAAAACTTCTCCACAACCC

CXCL9-S: TCTCCCAATTCATCCTCACTC

CXCL9-A: AATGGTCTGGTTGCCATCCT

CXCL10-S: ATGCAGTGCTTCCAAGGATG

CXCL10-A: ACCCCAAAGCAGAAAGATTCC

CXCL11-S: TTGGCTGTGATATTGTGTGCT

CXCL11-A: TGCTCTTTTCCAGGACTTCA

CXCL12-S: TGTGGCACTCAGATACCGACT

CXCL12-A: AAAGACGGATCTCACAGAGGG

CXCL13-S: TTCTTCACTCACAGCACCCTA

CXCL13-A: ATGGATTCCTTTGCCTCTTG

CXCL14-S: CATTTTATAGCTGCGTGCGAA

CXCL14-A: TGGAGTCACACTGAGGCTGT

CXCL16-S: AGAAGCAGCCGGAAAAAAA

CXCL16-A: TGACGCCTATAATCCTCGCA

CXCL17-S: GTGCAAAGATTGGTTCCTGA

CXCL17-A: TGATTTAGGGGTGGGTACAGT

Fibronectin-S: ATGTCTTGGGAACGGAAAAG

Fibronectin-A:TCTCGGGAATCTTCTCTGTCA

α-SMA-S: GACGAAGCACAGAGCAAAAGA

α-SMA-A: ATAGATGGGGACATTGTGGGT

PDGFβ-R-S: TAGCAAGTGCCTGTGTCCCT

PDGFβ-R-A: AAATGTGCAACCACCTGGAA

VE-Cadherin-S: TAGCCCTGCTCCAACTCCATA

VE-Cadherin-A: TCAAAGCAAGGTCTCAGCTCT

### Immunoblot

Cells (1–2 × 10^6^) were lysed in 200 ml lysis buffer (20 mM Tris, pH 7.5, 150 mM NaCl, 1% Triton X-100, 1 mM EDTA, 1 mM sodium pyrophosphate, 1 mM β-glycerophosphate, 1 mM Na_3_VO_4_, 1 mg/ml leupeptin). The cell lysate was centrifuged at 12,000 g at 4 °C for 5 min. Proteins were electrophoresed on 10% SDS-PAGE gels, and transferred onto Immobilon P membranes (Millipore, Billerica, MA, USA). The membranes were blocked by incubation in 3% nonfat dry milk for 1 h at room temperature and then incubated with primary antibodies in PBS containing 0.01% Tween 20 overnight at 4 °C. After incubation with a horseradish peroxidase-conjugated secondary antibody, the protein bands were detected with SuperSignal chemiluminescent substrate-stable peroxide solution (Pierce Rockford, IL, USA) and BIOMAX-MR film (Eastman Kodak Co., Rochester, NY, USA). When necessary, the membranes were stripped with Restore Western blot stripping buffer (Pierce) and re-probed with antibodies against various cellular proteins.

### Quantitative real time RT-PCR (qRT-PCR)

The qRT-PCR was performed as described by Sun *et al*.^49^. Briefly, total RNA was isolated and reverse transcribed as described above. The cDNA was amplified using TaqMan Universal PCR master mix (Roche Applied Science) and a LightCycle^®^ 96 detection system (Roche Applied Science). The amplification of the target genes was normalized using the amplification levels of glyceraldehyde-3-phosphate dehydrogenase (*GAPDH*) as an endogenous control. The efficiency of the PCR was tested by amplification of the target from serially diluted cDNA generated from the reverse transcription of a stock set of human RNA. The data analysis and calculations were performed using the 2^−ΔΔ*CT*^ comparative method, as described by the manufacturer. Gene expression is shown as the fold induction of a gene measured in IL-1-treated samples relative to samples cultured with medium. Same primes were used as described in RT-PCR.

### Enzyme-linked immunosorbent assay (ELISA)

The production of CXCL1, 2, and 8 in culture supernatants was detected by enzyme-linked immunosorbent assay (ELISA) according to the manufacturers’ standard protocols.

### Plasmid transfection

Cells, cultured in six-well plates, were transfected with 1 μg plasmid containing sequence coding for human A20 protein using Lipofectamine^TM^ 2000 (Invitrogen) according to the manufacturer’s instructions. Expression of A20 in the transfected cells was examined by western blot 48 h after transfection. For stable transfection, G418-resistant cells were selected after incubation with 800 μg/ml G418 for 3 weeks.

### siRNA transfection

siRNA against human A20 (sc-37655) and silencer negative siRNA control (sc-37007) were purchased from Santa Cruz Biotechnology (Santa Cruz, CA, USA). siRNA transfection reagent (sc-29528, Santa Cruz, CA, USA) was used to transfect siRNA into HMC cells according to the manufacturer’s instructions. Briefly, 0.25–1 μg siRNA and 6 μl siRNA transfection reagent were used for each transfection (6-well plates, 0.5 × 10^6^ cells/well). The knockdown of A20 was analyzed 48 h after siRNA transfection by western blot.

### Statistical analysis

All experiments were performed at least three times, and the representative results were shown. The results were expressed as the mean ± S.D. Differences between groups were examined for statistical significance using two tailed Student’s *t* test, and p values equal to or less than 0.05 were considered statistically significant (n = 3 for each qRT-PCR and ELISA test).

## Additional Information

**How to cite this article**: Luo, H. *et al*. A20 regulates IL-1-induced tolerant production of CXC chemokines in human mesangial cells via inhibition of MAPK signaling. *Sci. Rep*. **5**, 18007; doi: 10.1038/srep18007 (2015).
